# RETRACTED: Haghbin et al. Enhancement of the Electrical Conductivity and Interlaminar Shear Strength of CNT/GFRP Hierarchical Composite Using an Electrophoretic Deposition Technique. *Materials* 2017, *10*, 1120

**DOI:** 10.3390/ma19091843

**Published:** 2026-04-30

**Authors:** Amin Haghbin, Gholamhossein Liaghat, Homayoun Hadavinia, Amir Masoud Arabi, Mohammad Hossein Pol

**Affiliations:** 1 Faculty of Mechanical Engineering, Tarbiat Modares University, Jalal Ale Ahmad Highway, Tehran 14115-111, Iran; 2 Faculty of Science, Engineering and Computing, School of Engineering, Kingston University, London SW15 3DW, UK; h.hadavinia@kingston.ac.uk; 3 Department of Inorganic Pigments and Glazes, Institute for Color Science and Technology, Tehran 16688-36471, Iran; 4 Department of Mechanical Engineering, Tafresh University, Tafresh 39518-79611, Iran

The journal retracts the article titled “Enhancement of the Electrical Conductivity and Interlaminar Shear Strength of CNT/GFRP Hierarchical Composite Using an Electrophoretic Deposition Technique” [1], cited above.

Following publication, concerns were brought to the attention of the Editorial Office regarding an overlap between this [1] and an earlier publication [2] originating from a similar authorship group and published in another language.

In accordance with the journal’s standard procedures, the Editorial Office and Editorial Board conducted an investigation that confirmed a significant content duplication between the two above mentioned publications [1,2]. In light of the absence of appropriate disclosure at submission, including proper acknowledgement, citation, and clarification of copyright permission as required by the publisher’s policies, the Editorial Board have decided to retract this paper as per MDPI’s retraction policy. 

This retraction was approved by the Editor-in-Chief of the journal *Materials*.

The authors did not provide a comment on this decision.

## References

[1] Haghbin A., Liaghat G., Hadavinia H., Arabi A.M., Pol M.H. (2017). RETRACTED: Enhancement of the Electrical Conductivity and Interlaminar Shear Strength of CNT/GFRP Hierarchical Composite Using an Electrophoretic Deposition Technique. Materials.

[2] Haghbin A., Liaghat G., Arabi A.M., Pol M.H. (2017). Improving shear strength in nanocomposites through electrophoretic deposition of carbon nanotubes. Modares Mech. Eng..

